# P-1700. Benchmarking Clinical Outcomes by Antimicrobial Spectrum in Patients with Community-Acquired Pneumonia

**DOI:** 10.1093/ofid/ofae631.1866

**Published:** 2025-01-29

**Authors:** Kirthana Beaulac, Philippe Mentler, Jerod Nagel, Michael Postelnick, Pratish C Patel, Christopher McCoy, Patrick M Kinn, Danielle Casaus, Ethan A Smith

**Affiliations:** Emerson Hospital, Concord, Massachusetts; Vizient, Irving, Texas; Michigan Medicine, Ann Arbor, Michigan; Northwestern Medicine, Lake in the Hills, Illinois; Vanderbilt University Medical Center, Nashville, Tennessee; Beth Israel Deaconess Medical Center, Boston, Massachusetts; University of Iowa Hospitals & Clinics, Iowa City, Iowa; University of Kentucky HealthCare, Lexington, Kentucky; Ronald Reagan UCLA Medical Center, Los Angeles, California

## Abstract

**Background:**

Antimicrobial benchmarking provides insight into practice variation relative to peers and outcomes relative to projections. Incorporation of patient-level risk-adjustment amplifies the validity and utility of data generated. The Vizient Clinical Data Base (CDB) is a healthcare analytics platform incorporating granular patient-level data from over 1,100 US hospitals and can be used to benchmark patient outcomes. The CDB’s multi-variate 2023 Academic Medical Center Risk Model is validated to produce reliable estimates for patient outcomes.

**Figure:**
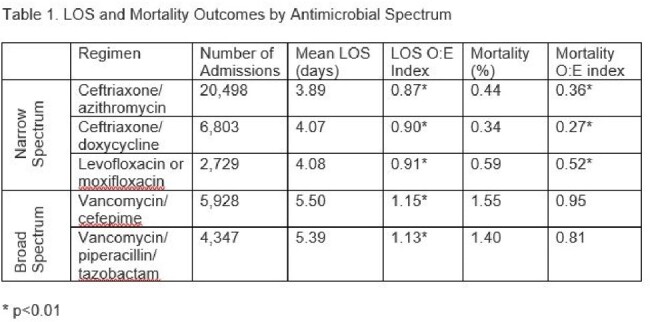

**Methods:**

The CDB evaluated length of stay (LOS) and mortality for hospital admissions for community-acquired pneumonia (CAP). Admissions in CY2023 were included when labeled with a principal ICD-10 code for bacterial pneumonia, adjudicated as present on admission, and when the admission source was an outpatient location. Compared to the observed LOS, the risk model computed expected LOS (eLOS) and produced an observed to expected (O:E) index; a similar O:E index was created for mortality. Those who received narrow-spectrum (NS) antimicrobials, defined as ceftriaxone plus either azithromycin (CA) or doxycycline (CD), or levofloxacin or moxifloxacin (FLQ) monotherapy, were compared to patients who received broad-spectrum (BS) therapy, defined as vancomycin plus either piperacillin/tazobactam (VPT) or cefepime (VCP). Admissions attributed to the NS cohort did not receive any doses of a BS (VPT/VCP) antimicrobial and vice versa. Admissions were limited to those with eLOS of 3-7 days to balance medical complexity between NS and BS cohorts and limit confounding amongst eLOS, morality estimates, and antimicrobial prescribing.

**Results:**

A principal diagnosis of CAP was assigned to 46,585 admissions. The LOS index was < 1 for NS regimens, but > 1 for those receiving BS therapy (Table 1). All deviations were statistically significant. The mortality index was < 1 for all antimicrobial combinations; however, it was significantly lower than expected (p < 0.01) for NS regimens (CA 0.36; CD 0.27; FLQ 0.52), but not for VPT 0.81 or VCP 0.95 (p > 0.05).

**Conclusion:**

After risk-adjusting for patient-level confounders impacting LOS and mortality, the use of NS antimicrobials for the treatment of CAP was associated with better outcomes than BS antimicrobials.

**Disclosures:**

**Kirthana Beaulac, PharmD**, Abbvie: Honoraria **Pratish C. Patel, PharmD, BCIDP, AAHIVP**, VBI Vaccines: Stocks/Bonds (Public Company)

